# BCMA-Targeting Therapy: Driving a New Era of Immunotherapy in Multiple Myeloma

**DOI:** 10.3390/cancers12061473

**Published:** 2020-06-05

**Authors:** Shih-Feng Cho, Liang Lin, Lijie Xing, Yuyin Li, Tengteng Yu, Kenneth C Anderson, Yu-Tzu Tai

**Affiliations:** 1 LeBow Institute for Myeloma Therapeutics and Jerome Lipper Multiple Myeloma Center, Dana-Farber Cancer Institute, Harvard Medical School, Boston, MA 02138, USA; sifong96@gmail.com (S.-F.C.); Liang_Lin@DFCI.HARVARD.EDU (L.L.); Lijie_Xing@DFCI.HARVARD.EDU (L.X.); Yuyin_Li@DFCI.HARVARD.EDU (Y.L.); Tengteng_Yu@DFCI.HARVARD.EDU (T.Y.); Kenneth_Anderson@dfci.harvard.edu (K.C.A.); 2 Division of Hematology & Oncology, Department of Internal Medicine, Kaohsiung Medical University Hospital, Kaohsiung Medical University, Kaohsiung 80708, Taiwan; 3 Faculty of Medicine, College of Medicine, Kaohsiung Medical University, Kaohsiung 80708, Taiwan

**Keywords:** multiple myeloma, MM, targeted immunotherapy, B-cell maturation antigen, BCMA, tumor targeting, tumor-associated antigen, monoclonal antibody, MoAb, chimeric antigen receptor T cell, CAR T, bispecific T cell engager, BiTE, antibody drug conjugate, ADC, bone marrow, BM, T cell dependent cytotoxicity, TDCC, antibody-dependent cellular cytotoxicity, ADCC, antibody-dependent cellular phagocytosis, ADCP, natural killer cell, NK cell, signal transduction

## Abstract

The treatment of multiple myeloma (MM) has entered into a new era of immunotherapy. Novel immunotherapies will significantly improve patient outcome via simultaneously targeting malignant plasma cell (PC) and reversing immunocompromised bone marrow (BM) microenvironment. B-cell maturation antigen (BCMA), selectively expressed in PCs and a key receptor for A proliferation-inducing ligand (APRIL), is highly expressed in MM cells from patients at all stages. The APRIL/BCMA signal cascades promote the survival and drug resistance of MM cells and further modulate immunosuppressive BM milieu. Impressively, anti-BCMA immunotherapeutic reagents, including chimeric antigen receptor (CAR), antibody-drug conjugate (ADC) and bispecific T cell engager (BiTE) have all shown high response rates in their first clinical trials in relapse and refractory patients with very limited treatment options. These results rapidly inspired numerous development of next-generation anti-BCMA biotherapeutics, i.e., bispecific molecule, bispecific or trispecific antibodies, a novel form of CAR T/NK cells and T Cell Antigen Coupler (TAC) receptors, antibody-coupled T cell receptor (ACTR) as well as a cancer vaccine. We here highlight seminal preclinical and clinical studies on novel BCMA-based immunotherapies as effective monotherapy and discuss their potential in combination with current anti-MM and novel checkpoint drugs in earlier disease stages to further achieve durable responses in patients.

## 1. Introduction

Multiple myeloma (MM), the second most hematologic malignancy, is characterized by excessive growth of malignant plasma cells in the bone marrow (BM), excessive production of monoclonal immunoglobulin, osteolytic bone lesions, impaired renal function, and immunosuppression [1]. The development of novel therapies incorporating proteasome inhibitors (PIs) and immunomodulatory drugs (IMiDs, i.e., lenalidomide, pomalidomide) have significantly improved the prognosis and survival of patients with MM for the last two decades. Therapeutic monocloncal antibodies (MoAbs) targeting CD38 (daratumumab, isatuximab) and SLAMF7 (elotuzumab) further improved outcomes of patients with relapsed/refractory MM (RRMM) [2,3,4,5]. Specifically, daratumumab and elotuzumab were approved by US Food and Drug Administration (FDA) in combination with lenalidomide and dexamethasone for the treatment of RRMM in 2015. In early March 2020, the second anti-CD38 MoAb isatuximab was approved in combination with pomalidomide and dexamethasone in patients who have received at least two prior therapies including lenalidomide and a PI. However, MM remains an almost incurable disease. The overall survival (OS) is extremely low in patients with relapses after treatment with PIs and IMiDs [6,7]. Thus, there remains urgent need to develop new therapeutics targeting different mechanisms and with superior potency to overcome drug resistance and minimize the risk of relapse.

**Figure 1:**
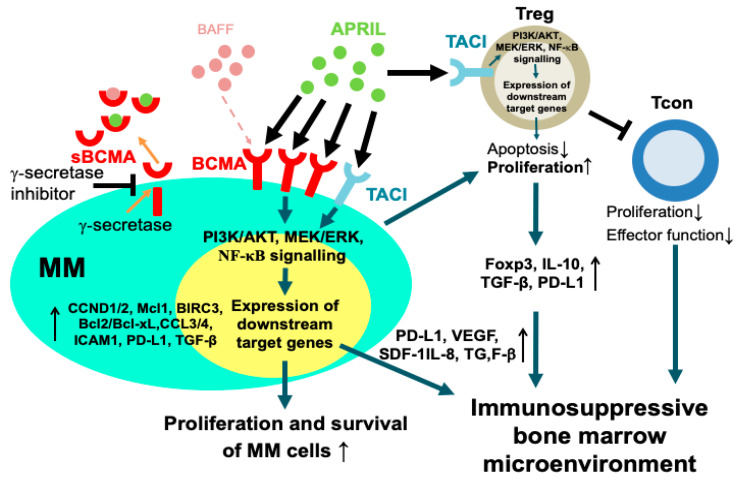
The A-proliferation inducing ligand/B cell maturation antigen (APRIL/BCMA) pathway and its biological effect in the multiple myeloma bone marrow microenvironment. APRIL is mainly secreted from macrophages and osteoclasts in the bone marrow (BM) microenvironment. It binds to BCMA with significantly higher affinity than B-cell activation factor (BAFF) to critically regulate multiple myeloma (MM) cell growth, survival, and drug resistance. BCMA is expressed at significantly higher levels than transmembrane activator and calcium modulator and cyclophilin ligand interactor (TACI) in MM cells to constitutively transmit tumor-promoting signaling. In addition to upregulate key cell cycle progression (i.e., CCND1/2), anti-apoptotic proteins (i.e., Mcl1, Bcl-2, Bcl-xL, BIRC3), osteoclast-promoting factors (i.e., CCL3/4, SDF-1), and adhesion molecules (i.e., ICAM-1, CD44), APRIL/BCMA signaling cascade further induces major immunosuppressive factors (i.e., IL-10, PD-L1, TGF-β, VEGF) in MM cells. BCMA on the cell membrane of MM cells is shed by γ-secretase and soluble BCMA (sBCMA) is detected in serum samples of MM patients. Inhibitors blocking cleavage by γ-secretase can reduce the generation of sBCMA. MM cells further stimulate proliferation of regulatory T cells (Treg) via cell–cell contact and cytokine factor-dependent mechanisms. Despite no BCMA expression, regulatory T cells (Treg) utilizes TACI to deliver APRIL signaling pathway while conventional T cells (Tcon) rarely express TACI when compared with Treg. The interaction of APRIL with TACI induces the expression of proliferation and survival genes as well as central immunosuppressive markers (Foxp3, IL-10, PD-L1, TGF-β) in Treg but not Tcon. This signaling pathway enhances the inhibitory effects of Treg on conventional T cells (Tcon), thereby decreasing the proliferation and function of Tcon. Significantly, these signaling pathways contribute to the pathophysiology of MM cells and MM-induced immune suppression in the BM microenvironment.

The identification and validation of specific MM antigens are crucial in developing effective targeted immunotherapies for MM. B-cell maturation antigen (BCMA), also termed tumor necrosis factor receptor superfamily member 17 (TNFRS17) or CD269, is a type III transmembrane protein containing cysteine-rich extracellular domains [8,9]. BCMA, together with B-cell activation factor receptor (BAFF-R) and transmembrane activator and calcium modulator and cyclophilin ligand interactor (TACI), are critical regulators during the maturation and differentiation of B-cells into plasma cells (PCs). Among them, BCMA is selectively expressed from the late stage of B-cell maturation to terminal differentiation of antibody-producing PCs, concomitantly with the loss of BAFF-R. These three functionally related receptors contribute to the long-term survival of B-cell during its development by binding to BAFF and/or A proliferation-inducing ligand (APRIL) [10,11,12]. In MM, BAFF supports MM cell adhesion and survival via a paracrine mechanism [13,14]. Compared with BAFF, APRIL does not bind to BAFF-R and is predominantly produced by MM-supporting BM accessory cells, i.e., macrophages, osteoclasts, and other myeloid lineage cells [15,16,17,18,19]. APRIL also binds to BCMA with a significantly higher affinity (>2-log) [12] and specificity than BAFF, and promotes MM cell growth and survival in vivo [19,20]. The APRIL/BCMA signaling pathway supports drug resistance of MM cells [15,19,21] and immunosuppressive MM BM microenvironment via direct induction of key downstream anti-apoptotic genes (Mcl-1, Bcl-2/Bcl-xL) and immune regulatory genes (IL-10, PD-L1, VEGF, TGF-β) in MM cells [18,19,22]. Moreover, APRIL binding to TACI induces anti-apoptotic and immune inhibitory factors in MM cells and myeloma-promoting regulatory T (Treg) cells [15,19,23,24,25] (Figure 1). 

Analysis of patient samples from various cohorts demonstrated that BCMA mRNA and protein levels are highly and specifically expressed in PCs but no other normal tissues [26,27,28,29,30,31]. Besides PCs, only plasmacytoid dendritic cells which protect MM cell growth and survival have detectable BCMA transcript and protein, but its levels are significantly lower in these cells when compared with in paired MM cells from the same patients [28]. BCMA expression is significantly elevated in malignant vs. normal PCs and throughout disease progression. Since BCMA is a substrate for gamma-secretase, the extracellular domain of BCMA is shed [32]. Soluble BCMA (sBCMA) is detected in patient sera and supernatants from MM cell culture [29,33]. Soluble BCMA may act as a decoy and prevent BAFF from binding to membrane-bound BCMA to inhibit normal B-cell differentiation to PCs [34]. In addition, MM patients have higher levels of surface BCMA expression and sBCMA than health individuals [33], in accordance with increased BCMA transcript levels in patient MM cells vs. normal PCs of healthy donors [26,28]. Levels of BCMA transcript and proteins, either membrane or soluble forms, were not affected by certain anti-MM treatment, including PIs or IMiDs. Furthermore, higher sBCMA levels in patient serum is associated with higher MM disease burden and poorer clinical outcome [33,35,36]. 

Because of its significant pathophysiologic and clinical relevance, BCMA holds great promise for targeted immunotherapy in MM.

### 1.1. BCMA Is an Excellent Target for Anti-MM Immunotherapy: Comparison with the Other Therapeutic Targets

In MM, several surface molecules or molecular pathways are druggable and targeted, including CD38, CD56, SLAMF7, CD138, programmed cell death-ligand 1(PD-L1), and BCMA [19,37,38,39]. Anti-CD38 MoAbs effectively eradicate MM cells and induce immunomodulatory effects to potentially restore immune effector cell function and mitigate immunosuppressive cells [40,41,42,43]. Like anti-CD38, anti-SLAMF7 MoAb elotuzumab induces antibody-dependent cellular cytotoxicity (ADCC) against MM cells mediated by natural-killer (NK) cells [37]. However, normal key immune effector cells (NK, T) and certain hematopoietic cells, especially when activated, also express CD38, CD56, or SLAMF7 on their surface [44,45]. 

Program cell death 1 (PD1)/PD-Ligand 1(PD-L1) immune checkpoint pathway is another druggable target for MM immunotherapy and IMiDs enhanced the anti-MM activity of anti-PD1 or anti-PD-L1 MoAbs in a preclinical study [38]. However, in a phase 1 trial investigating nivolumab monotherapy in 27 RRMM patients, no significant objective response was observed [46]. Although a combination of pembrolizumab with lenalidomide and dexamethasone showed higher overall response rate (ORR) (50%) [47], the phase 3 studies which combined IMiDs with PD1 or PD-L1 inhibitors were stopped due to high toxicity and the high risk of mortalities which may be caused by uncontrolled immunoreactivity [48].

Other novel antigens are also explored for MM immunotherapy. For example, the orphan G protein-coupled receptor, class C group 5 member D (GPRC5D), which is mainly expressed on CD138+ MM and hair follicular cells [49]. Preclinical studies showed that combined anti-BCMA and anti-GPRC5D CAR-T may further eradicate MM cells and reduce risk of relapse due to low or lost BCMA [49,50]. Activated Integrin β 7 (ITGB7) is widely expressed on MM cells but not on other cells [51]. The preclinical study which constructed CAR-T cell targeting ITGB7 also showed significant anti-MM activity without causing damage to normal hematopoietic cells. Other antigens for anti-MM CAR-T therapy include SLAMF7 [45], CD38 [52] and CD229 [53].

BCMA shows its superiority based on its specific expression in PCs, plasmacytoid dendritic cells, plasmablasts, and mature PCs, but not in earlier phase and memory B cells, hematopoietic cells and other normal tissue cells [28,29,30,54]. The APRIL/BCMA pathway in the pathophysiology of MM was also validated in mice models [19,20]. Furthermore, anti-BCMA antibody was detected after successful donor lymphocyte infusion in relapsed MM patients after allogeneic stem cell transplant, suggesting BCMA as a target of donor B-cell immunity [55]. Based on these unique characters, BCMA is an ideal druggable target for anti-MM immunotherapy with minimal off-target toxicity in other normal tissues.

### 1.2. Anti-BCMA Immunotherapy: From Bench to Bedside and Back to Bench Studies

The breakthrough of anti-BCMA immunotherapy in MM was reported in preclinical studies on the first chimeric antigen receptor (CAR) T cells in 2013 [29] and the anti-BCMA J6M0 with engineered Fc conjugated with MMAF via uncleavable linker in 2014 [28]. These two studies confirmed very restrictive BCMA expression at both transcript and protein levels in MM PCs but no other normal tissues. Significantly, both anti-BCMA CAR T-cells and J6M0 MMAF ADC were highly active to kill patient MM cells in vitro and in vivo [28,29,56]. From then on, several BCMA-based treatment modalities, including novel chimeric antigen receptor (CAR) T cell therapy, antibody-drug conjugate (ADC), bispecific T-cell engager (BiTE), bispecific molecule, and bi/tri-specific antibodies as well as Antibody-Coupled T-Cell receptor (ACTR) [57] and T-cell antigen coupler (TAC) [58] are under development and investigation [22,25,59,60]. 

## 2. Immunotherapies Targeting BCMA

### 2.1. CAR T-Cell Therapy

CAR T cell therapy, independent of major histocompatibility complex (MHC) restriction, is characterized by genetically modified T cells to induce powerful anti-tumor cytotoxic ability via high specificity targeting tumor antigen [61]. In 2017, the first CAR T-cell therapy was approved by the FDA in CD19+ acute lymphoblastic leukemia (ALL) [62]. In MM, promising early results reported in late 2016 quickly led to two BCMA CAR T therapies bb2121/Idecabtagene vicleucel and P-BCMA-101 for Breakthrough Therapy Designation and Regenerative Medicine Advanced Therapy (RMAT), respectively, granted by US FDA, by late 2018 [25,59]. Table 1 and the following section highlight impressive clinical responses reported from various trials thus far. 

ASCT, Autologous stem cell transplant; BM, bone marrow; Cy, cyclophosphamide; CNS, central nervous system; CR, complete response; CRS, cytokine releasing syndrome; DFS, disease-free survival; DLT, dose-limiting toxicity; DOR, duration of response; EGFR, epidermal growth factor receptor; EFS, event-free survival; EM, extramedullary; FCM, flow cytometry; Flu, fludarabine; IRR, infusion related reaction; MoAb, monoclonal antibody; MTD, maximum tolerated dose; MR, minimal response; MRD, minimal residual disease; MRD-, MRD-negative; NR, not reached; OS, overall survival; PCL, plasma cell leukemia; PD, progressive disease; PFS, progression-free survival; PR, partial response; RRMM, relapsed and refractory multiple myeloma; scFv, single-chain variable fragment; sCR, stringent complete response; Tscm, T stem cell memory phenotype; VGPR, very good partial response; WM, Waldenström’s macroglobulinemia.

#### New CAR T Therapy Strategies

Newer CAR T cells with modified CAR structure are under rigorous investigation to improve the efficacy and optimization of production protocols to achieve cost and time effectiveness as well as durability of cells. A 3rd generation anti-BCMA CAR was generated with fully human BCMA scFv and 4-1BB, CD3ζ signaling domains and tEGFR safety switch [67]. After conditioning therapy, such CAR T-cells composed of a 1:1 ratio of CD4+:CD8+ cells were infused in RRMM patients. In another clinical study reporting 80% ORR, anti-CD19 and anti-BCMA CAR T cells were infused in RRMM patients after conditioning therapy [83]. Importantly, a patient with progression disease with presentation of extramedullary lesion after CAR T cell infusion achieved remission after local injection of anti-BCMA CAR T cells. Moreover, a new CAR-T therapy was reported as a consolidation treatment after autologous transplantation [70]. Newly diagnosed stage III MM patients who failed to achieve partial response (PR) after induction received anti-CD19 and BCMA CAR-T therapy 14 to 20 days after transplantation. The ORR was 100% in nine evaluable patients, including 3 complete response (CRs) and 6 very good partial response (VGPRs). MRD negativity increased from 37.5% after transplantation to 66.7% after CAR-T therapy. Furthermore, clinical investigations are ongoing for other BCMA CAR-T treatment approaches.

### 2.2. Antibody-Drug Conjugate (ADC) Studies

ADC is composed of a therapeutic MoAb and a potent cytotoxic chemicals (payload), which are covalently connected via a synthetic chemical linker [84]. After administration of ADC, the MoAb first identifies and binds to the tumor antigen on the tumor cells, and then is internalized with the payload. Inside the tumor cell, the cytotoxic chemicals are released and specifically kills the tumor cells. This novel drug class aims to maximize tumor cell death while minimize unspecific toxicity to allow for a favorable therapeutic window. Currently, several anti-BCMA ADCs for MM treatment are under development and two ADCs delivering different payloads have entered into clinical investigation. GSK2857916 (Belantamab mafodotin) have received FDA Breakthrough Therapy Designation status in 2018.

#### 2.2.1. GSK2857916 (Belantamab Mafodotin) (GlaxoSmithKline) 

GSK2857916, the first anti-BCMA ADC entering clinical study, is composed of a humanized IgG1 mAb with increased affinity to effector cells due to its dyfucosylated Fc and high affinity to BCMA (Kd: ~0.5 nM), a novel non-permeable anti-tubulin agent, MMAF payload, as well as a non-cleavable linker, maleimidocaproyl (mc) [28,56]. GSK285791 significantly and selectively kills MM cells via the direct inhibition of proliferation, induction of apoptosis of MM cells, as well as ADCC and antibody-dependent cellular phagocytosis (ADCP) in vitro and in vivo. GSK285791 rapidly eliminated MM cells with tumor-free survival up to 3.5 months in mice. The clinical studies of GSK2857916 (Belantamab mafodotin) are shown in Table 2.

#### 2.2.2. MEDI2228 (MedImmune LLC)

MEDI2228 is composed of a fully human antibody which specifically conjugates to a pyrrolobenzodiazepine (PBD) dimer via a protease-cleavable linker [91]. MEDI2228 significantly induced cytotoxicity against MM cell lines (IC_50_: 6–210 ng/mL) and quiescent myeloma precursor cells. Compared with its MMAF ADC homolog, MEDI2228 delivering PBD showed more potent cytotoxicity in patient MM cells and MM progenitor cells which are not proliferating [92]. Furthermore, MEDI1228 preferentially binds to membrane bound BCMA, thereby minimizing the inhibition of sBCMA on anti-BCMA mAb-induced anti-MM activity in vitro and in vivo.

Unlike its MMAF ADC homolog, MEDI2228 triggered DNA damage response (DDR) via phosphorylation of ATM/ATR kinases, CHK1/2, CDK1/2, and H2AX, further inducing DDR-related gene expression [92]. MEDI2228 induced synthetic lethality when combined with DDR inhibitors (DDRi s) targeting ATM/ATR/WEE1 checkpoints. Importantly, MEDI2228 and bortezomib combination enhanced apoptosis of drug-resistant MM cells and superior in vivo efficacy, further prolonging host survival than monotherapy via increased nuclear γH2AX-expressing micro-foci, irreversible DNA damages, and irreversible tumor cell death. A Phase 1 clinical study (NCT03489525) is ongoing in RRMM patients who are either post autologous stem cell transplant or transplant ineligible.

#### 2.2.3. HDP-101

HDP-1 is an antibody-targeted amanitin conjugate (ATAC), composed of compound of maleimide-amanitin conjugation and engineered cysteine residues in the heavy chain of the humanized anti-BCMA Thiomab [93,94]. Amanitin binds to the RNA polymerase II in eukaryotic cells and inhibits the cellular transcription process. In in vivo studies using mice or Cynomolgus monkeys, administration of HDP-101 resulted in significant tumor regression with good tolerability, therapeutic index, and long serum half-life of HDP-101 (about 12 days) [94,95].

### 2.3. Bispecific T-Cell Engager (BiTE) Molecules

BiTE^®^ is a small-sized molecule (55 kDa), which is a single-chain variable fragment (scFv) with two linked mAbs (bispecific antibodies), with one targeting mainly CD3 on T-cells and the other one targeting tumor-associated antigens on tumor cells [96,97]. BiTE^®^ molecules simultaneously link T-cells and tumor cells, leading to the formation of immune synapse followed by lysis of tumor cells and activation of T cells [98,99].

#### 2.3.1. BI 836909/AMG 420

BI 836909 is the first anti-BCMA BiTE® into preclinical and clinical investigation in MM [99]. BI 836909 consists of two linked scFvs, with one scFv targeting BCMA positioned in N-terminal and the other scFv targeting CD3ε in C-terminal (followed by a hexahistidine, His6 tag). In mouse xenograft studies, BI 836909 led to tumor shrinkage and prolonged host survival. The cynomolgus monkey study showed significant depletion of BCMA+ PCs in the BM after administration of BI 836909. The findings of BI 836909/AMG 420 clinical study are shown in Table 2. 

#### 2.3.2. AMG 701

AMG 701 is the novel form of anti-BCMA BiTE^®^ with an extended serum half-life to 112 h and demonstrated potent anti-MM activity in the preclinical studies [100]. AMG 701 further induced robust immunomodulatory effects, including the activation and proliferation of CD4 and CD8 T cells as well as differentiation of memory T cells [100]. Moreover, a combination of AMG 701 and IMiDs (lenalidomide or pomalidomide) enhanced anti-MM activity of AMG 701 and upregulated effector cell function to further prevent disease relapse in the SCID mouse model for human MM. A clinical trial for AMG 701 in RRMM (NCT03287908) is ongoing.

### 2.4. Bispecific or Trispecific Antibodies/Molecules

Currently, many bispecific Abs target BCMA on MM cells and CD3 on T cells or CD16 on NK effector cells. EM801 [30], BCMA-TCB2/EM901 (CC-93269) [30,89], JNJ-7957 [101] and TNB-383B [60,102,103] all target CD3 on T cells and BCMA on MM cells. AFM26, a tetravalent bispecific Ab targeting BCMA on MM cells and CD16A on NK cells, showed potent NK-cell-medicated ADCC [104]. HPN217, a BCMA-targeting tri-specific T-cell activating construct (TriTAC), simultaneously binds to MM cells, human serum albumin, and CD3 on T cells [104]. Engineering of a human albumin-binding domain into HPN217 extends serum half-life up to 3 weeks [105] (NCT04184050).

In 2019, the preliminary results were reported for two bispecific antibodies in early phase studies, CC-93269/EM901 (Bristol–Myers Squibb) and PF-06863135 (Pfizer) (Table 2).

### 2.5. Other Approaches

#### 2.5.1. Descartes-08 (Cartesian Therapeutics)

Descartes-08, RNA-generated anti-BCMA CD8 CAR T cells, showed CAR-specific suppression of myeloma maintained throughout the duration of treatment in a mouse model of disseminated human MM [106]. The magnitude of cytolytic and cytokine responses correlates with the duration of anti-BCMA CAR expression. This may be more cost-effective and decrease the risk of severe CRS. A trial of Descartes-08 in patients with RRMM (NCT03448978) is ongoing.

#### 2.5.2. Anti-BCMA Cancer Vaccine

In MM, cancer vaccines are generally considered to be a part of combination therapy together with other effective immunotherapies [25]. They are under early phase clinical investigation for high-risk smoldering MM (SMM) or post-autologous transplant setting in MM patients [107]. Engineered anti-BCMA peptides could increase affinity and stability to HLA-A2 and activate highly functional BCMA-specific cytotoxic T cells in vitro [25]. 

#### 2.5.3. Treatment Targeting APRIL/BCMA Pathway

Due to their impressive safety profile and significant APRIL-lowing effect by BION-1301, the first humanized MoAb against APRIL, in the Phase 1 and 2 study, a rationale was provided for further combination therapy with BCMA-based immunotherapy or other anti-MM agents [108].

#### 2.5.4. Antibody-Coupled T Cell Receptor (ACTR)

ACTR technology genetically engineers autologous T cell to express extracellular CD16 Fc receptor targeting NK cells and intracellular T cell signaling and costimulatory domains [109]. However, the clinical studies of ACTR087 with a 4-1BB-containing receptor combined with an anti-BCMA MoAb, SEA-BCMA in MM or other hematologic malignancy were halted due to safety concerns (NCT03266692) [57]. 

#### 2.5.5. T Cell Antigen Coupler (TAC) T Cell Therapy

TAC technology is a chimeric receptor that includes an antigen-binding domain, CD3ζ-binding domain, and a CD8/CD4 co-receptor [58]. TAC is cable of directing T cell receptor-CD3 complex towards a target antigen, leading to T cell activation in an MHC-independent fashion. Compared with conventional CAR, TAC co-opting endogenous TCR has higher anti-tumor activity, lower off-tumor effect and cytokine release, as well as no upregulation of immune checkpoint markers or T cell exhaustion. The BCMA TAC T cells rapidly eradicate MM cells in a murine model. Mice without tumors after TAC T cell treatment were resistant to the subsequent infusion of fresh tumor cells, suggesting the persistence of TAC T cells. 

## 3. Potential Biomarkers for BCMA-Based Immunotherapy

Immunotherapy targeting BCMA achieved a high response rate in clinical investigations. Currently, there are more BCMA CAR-T clinical trials than other anti-BCMA agents. Data published in these trials has provided valuable information for further clinical investigation for BCMA-based immunotherapies in MM.

Several parameters were demonstrated to be predictive for treatment response. First, several studies showed that the serum level of sBCMA decreased after successful CAR-T treatment. More significant changes in serum sBCMA level or BCMA expression on MM cells were seen in hematologic responders than in non-responders [36,63]. In the clinical trial investigating AMG 420, a rapid and sustained decrease in sBCMA level also suggested an early response [88]. Based on these finding, the change of sBCMA could be used to predict early response of BCMA-based therapy. However, more studies are needed to define the optimal fold-change in reduced sBCMA as a reliable indicator of therapeutic response.

In BCMA CAR-T studies, some parameters were also noted to be linked to treatment outcome. For example, a higher peak in CAR-T cell or total CAR-T cell expansion was associated with better clinical response [36,63,64]. Besides, a higher proportion of CD8+ T cells within the leukapheresis product with a CD45RO-CD27+ memory phenotype was significantly associated with peak CAR-T cell expansion and better response [36]. Other markers to predict disease progression include the appearance of anti-CAR T antibody and decreased residual CAR T+ cells [78]. 

MRD negativity status was utilized to evaluate the clinical efficacy of anti-BCMA immunotherapeutic in clinical trials [110]. A high MRD negativity rate was observed in some trials, especially in CAR-T treatment related studies. However, some patients with negative MRD still suffered from MM relapse or progression after successful CAR-T treatment, raising concerns regarding the limitations of current MRD detection tools. Currently, multi-color flow cytometry and next-generation sequencing are major tools to detect MRD in these trials. Both techniques are characterized by high sensitivity to detect MRD. However, BM aspiration is required for both methods. The patchy nature of MM cells in the BM may lead to a false negative result, thereby fail to identify some relapsed patients presented with an extramedullary disease. To address this issue, the incorporation of novel imagine-based methods, including whole-body positron emission tomography or magnetic resonance imaging, may reduce this risk [110]. Moreover, the development of novel liquid biopsy analyzing circulating tumor cells or cell-free DNA may further optimize MRD assessment.

## 4. Which Anti-BCMA Agent? Each Has Its Own Merits

Preclinical and clinical studies to date have indicated that BCMA is a promising molecule for disease monitoring and confirmed that BCMA is an important target antigen for immunotherapy in MM. Several ongoing Phase 2/3 studies continue to show the efficacy of CAR T therapy in RRMM [25,59,60]. Despite a high RR (≥70–100%) and increased survival by CAR-T cell therapy in the heavily pretreated RRMM patients who have no treatment options left [111], the high incidence of CRS and neurotoxicity, as well as the toxicity of conditioning chemotherapy are major concerns when selecting patients for this therapy (Table 3). In patients with high tumor burden, the risk of CRS is even higher, resulting in exclusion from clinical studies [63]. Moreover, approximately 30% MM patients are older than 75 years and may not be physically fit for potent conditioning chemotherapy and CAR-T cell production [112]. Besides, current manufacturing process to generate CAR-T cells requires to first collect patient T cells and it can take from 10 days to two weeks to complete the whole procedure. This could hold up the treatment for patients with a rapidly progressing disease. We should consider patients’ performance, disease status, and bridging or salvage therapy when using CAR-T cell therapy for RRMM patients in real world clinical practice. 

Meanwhile, the role of CAR-T therapy as consolidation therapy in high-risk MM patients after major treatment is under investigation. As mentioned above, one clinical trial evaluating CD19 and BCMA CAR-T cell therapy after tandem transplant, demonstrated that CAR-T cell therapy further improved treatment response with acceptable safety [70]. The long-term follow-up result is pending. Moreover, it is still an open question whether CAR-T therapy can replace conventional ASCT in newly diagnosed high-risk MM patients. When CAR-T cell therapy can be widely performed, we may expect a direct comparison to answer this question.

Like CAR-T therapy, the anti-MM mechanism of BiTE and bispecific Abs/molecules are also mainly via TDCC. The historic data and current AMG 420 study suggested that BiTE might have a better safety profile regarding to severe CRS and neurotoxicity, compared with CAR T therapy, especially in patients with a large tumor burden [113]. Furthermore, BiTE and bi-/tri-specific Abs/molecules are “off-the shell” products and could be immediately administered in patients with rapid progressed disease [114]. 

The therapeutic potential of these agents for MM patients in different disease stages are currently (or will be) tested. The decision should be made individually as to which forms of BCMA-based drugs should be used. According to the available published and historical data of CD19 BiTE^®^ Blinatumomab for ALL treatment, BiTE would bring better clinical benefit in patients who received less prior lines of treatment, since the T effector cell function would be better reserved (or less exhausted) than patients with heavier prior treatments [115,116]. For the RRMM patients who have received multiple lines of prior treatments, the function of immune cells would be profoundly compromised, therefore CAR-T therapy may be the better choice because CAR-T cells are activated during genetic modification process and these CAR T cell can further expand to develop persistent immunity after single infusion. Furthermore, CAR-T cell therapy also exerted potent anti-MM activity and induced significant tumor shrinkage in patients with extramedullary MM involvement [64,78]. 

Regarding the role of ADCs, these “off the shelf” products can also be used in patients with rapidly progressing disease due to their potent direct killing of MM cells without the need for patient effector cells. ADC could be more suitable in patients with a higher tumor burden since its AEs may be less- or unrelated to tumor burden, like CRS or the neurotoxicity observed in CAR-T therapy [63]. Anti-BCMA ADC can be used as major treatment or as a bridging therapy to quickly reduce tumor burden and allow for the recovery of patient immune effector T cells for subsequent CAR-T cell or BiTE therapy. 

## 5. Future Perspectives

**Figure 2:**
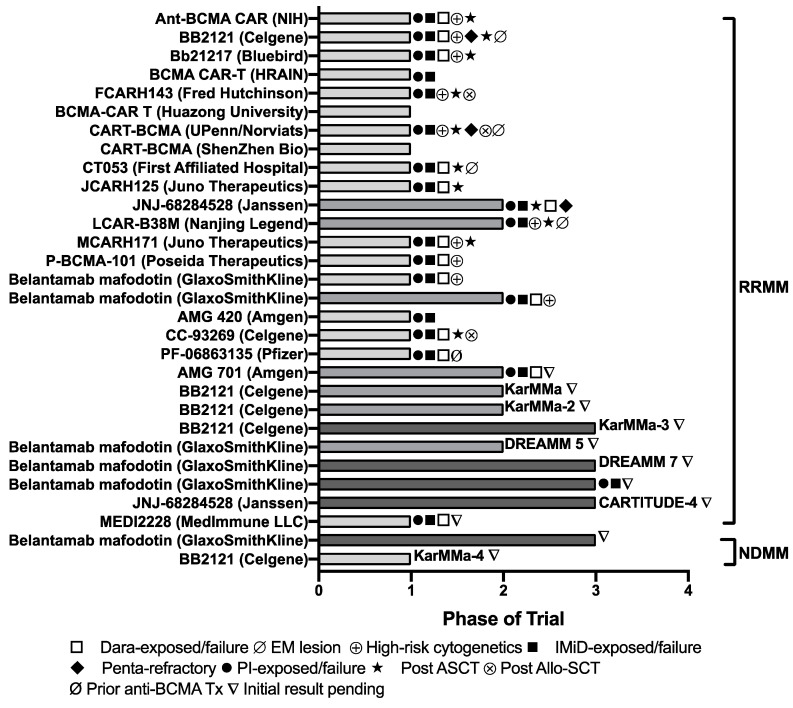
Immunotherapies targeting BCMA in clinical development. This figure shows various anti-BCMA agents in current clinical trials. The major enrollment criteria or characteristics of participants are also indicated. Every effort has been made to obtain reliable data from multiple sources including http://clinicaltrials.gov/, company, and other web sites, but accuracy cannot be fully guaranteed to date.

**Figure 3:**
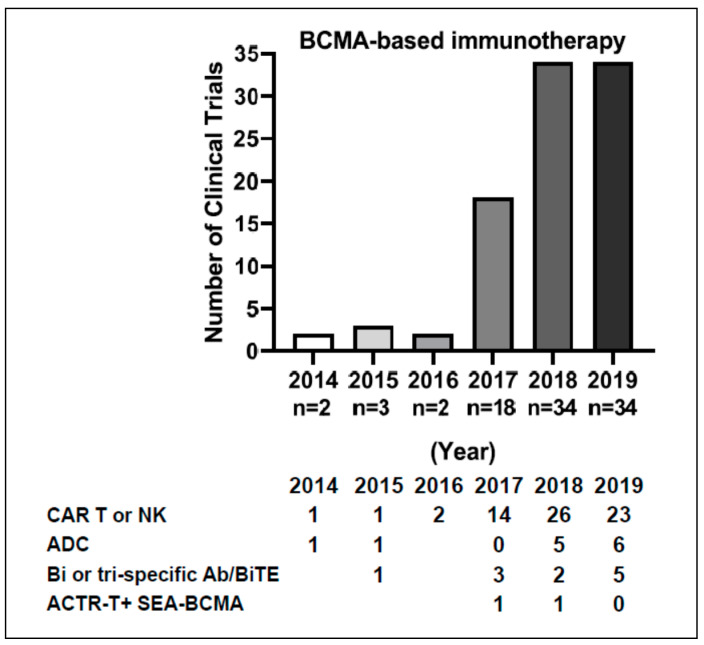
The annual number of clinical trials of BCMA-targeting immunotherapy in MM since 2014. The searching word is “myeloma”. The “Year” indicates the first post year of the trial on Clinicaltrial.gov. Shown below are the numbers of trials in various immunotherapeutic modalities each year. For example, there are 23 CAR T, 6 ADC, and 5 Bi/Tri-specific Ab/BiTE trials in 2019. Every effort has been made to obtain reliable data from multiple sources including http://clinicaltrials.gov/, company, and other web sites, but accuracy cannot be fully guaranteed to date.

Immunotherapy targeting BCMA has positioned itself in the treatment management for MM. Large trials are underway, including bb2121, P-BCMA-101 Tscm CAR-T cells, JNJ-68284528, and Belantamab mafodotin in RRMM patients, in KarMMa-3 (NCT03651128), NCT03288493, CARTITUDE-4 (NCT04181827), DREAMM 5 (NCT04126200), and DREAMM 7 (NCT04246047) as monotherapy and/or combination therapies (Figure 2). The number of registered clinical studies has increased significantly in the last 2 years (Figure 3). 

In parallel with new anti-BCMA therapy clinical trials, great efforts are making to further optimize BCMA-based immunotherapy. For CAR-T therapy, new scFv with a higher binding affinity to tumor antigen (like bb2121 or LCAR-B38M) may exert more potent anti-tumor activity. Using 4-1BB as a costimulatory domain can increase memory phenotype and enhance the persistence of CAR T cells after infusion. In addition, humanizing murine or fully humanized scFvs combined with optimized hinge and transmembrane domains may be less immunogenic with better safety profile [117,118]. A recent clinical trial demonstrated that the re-infusion of BCMA CAR-T cells with humanized scFv may overcome treatment failure due to impaired persistence by immune responses against murine scFv [74]. Furthermore, the addition of a safety switch or the development of inhibitory CAR as well as on-switch CAR may decrease the toxicity of CAR-T therapy [119,120]. Novel autologous mRNA-generated CAR T cells have a limited lifespan, which may reduce the risk of CRS and increase the treatment flexibility of multiple dosing [106] (NCT03448978). The addition of a gamma secretase inhibitor is also being explored to enhance membrane BCMA expression in order to increase CAR T efficacy (JSMD194 in NCT03502577) [121]. The preliminary data of the first clinical trial showed a high response rate (100%) and MRD negativity rate (83%) [122]. In addition, pretreatment of a gamma secretase inhibitor in low-BCMA expression patients can enhance BCMA expression and improve the treatment response of BCMA CAR-T therapy [123]. Since BCMA^low^ or BCMA^-^ relapse were observed in CAR-T trials, dual-targeting CAR or combined two CARs binding to BCMA and other MM-related antigens including CD19, CD138, and SLAMF7 are also under development, aiming to reduce the risk of low or lost BCMA expression-related relapse (antigen escape relapse) [59]. Other potential strategies include a faster ex vivo manufacturing process and allogeneic CAR-T like ALLO-715 (NCT04093596). Another potential study is to generate CAR-T cells using T cells collected earlier in the disease. Furthermore, NK-based cellular therapy may also achieve high treatment response but with a better safety profile than CAR-T therapy, since NK cells have a shorter lifespan than cytotoxic T cells (NCT03940833).

ADC Belantamab mafodotin has shown promising results in phase 1 and 2 trials, in which a high percentage of corneal events was also reported. The exact mechanism remains unknown, but it may be associated with the non-specific uptake of ADC with MMAF or other anti-microtubule cytotoxins as payload into actively dividing epithelial cells of cornea [124]. Ongoing strategies to further optimize BCMA ADC include novel payloads with different mechanisms of action like MEDI2228 (NCT03489525), new linkers to further avoid the premature release of drugs, and better design of an antibody like non-IgG scaffolds or non-internalizing MoAb scaffolds to enhance penetration [84].

Conventional BiTE^®^ molecule AMG 420 and similar bispecific antibody fragments have a common limitation, a relatively shorter half-life, making continuous infusion necessary to maintain adequate therapeutic serum level. Although the short half-life of these agents may help to handle treatment-related side effects like CRS, continuous infusion is also associated with a higher risk of catheter- or device-related infection [88]. To address this issue, several groups are investigating novel bispecific antibody constructs with an extended half-life or bispecific Abs in clinical trials with already-significant anti-MM activities reported in CC-93269 (NCT03486067) [89].

Despite a high RR in clinical trials, about half of patients receiving anti-BCMA treatment eventually relapse, indicating that treatment resistance remains a critical issue. Hypotheses include the confounding effect of sBCMA, antigen escape, and immunosuppressive BM microenvironment [59,103]. Strategies are being developed to overcome these issues. First, MEDI2228 preferentially binds to membrane vs. soluble BCMA [91]. The gamma-secretase inhibitor JSMD194, in the presence of sBCMA, may enhance the anti-MM effects of BCMA CAR T [121] (NCT03502577). Second, clinical trials of combination of Belantamab mafodotin with anti-PD1 MoAb or other standard-of-care MM agents are ongoing (NCT03848845, NCT03544281, and NCT03715478). Other combinations, such as AMG 701 plus IMiDs or MEDI2228 plus bortezomib also demonstrated impressive anti-MM activity in preclinical studies [92,100]. Third, for low or loss of BCMA expression on MM cells observed in relapsed patients after CAR-T therapy, the combination of anti-BCMA CAR-T cells with CAR targeting another MM antigen like CD38, CD138, SLAMF7, or CD19 may decrease the risk of antigen-escape-related treatment failure. The sequential infusion of CAR-T cells targeting CD19 and BCMA in RRMM patients has shown high RR [71]. 

To achieve the deepest response as early as possible, it is rational to next test these agents in frontline settings and earlier stages, i.e., newly diagnosed and SMM. CAR-T and BiTE therapy have already achieved a high MRD-negative rate in the sickest RRMM patients. Better preserved function of immune cells in SMM indicates that anti-BCMA immunotherapies will be more efficacious, and thereby may even prevent disease progression. A Phase 2 trial has begun in evaluating bortezomib, lenalidomide, and dexamethasone with or without anti-BCMA ADC belantamab mafodotin in newly diagnosed and transplant ineligible MM patients (NCT04091126). Another single arm study is also underway to determine the optimal target dose and safety of bb2121 in high-risk newly diagnosed MM patients (KarMMa-4 trial; NCT04196491).

## 6. Conclusions

The unprecedented clinical results of BCMA-based immunotherapies are impacting the treatment landscape in MM. Optimal clinical benefit will be achieved by their use in combination or in sequence, as well as treatment in earlier stages. BCMA-based immunotherapies will continue to transform the treatment management and improve patient outcome in MM for years to come.

## Figures and Tables

**Table 1:** Summary of early phase clinical trials of anti-BCMA CAR T-cell therapy.

Name (Sponsor)	Structure	Phase	Key Inclusion Criteria (Summarized)	Key Exclusion Criteria (Summarized)	Basic Data of Study Population	Protocol		Efficacy				Adverse Events (AEs)	
						Condi-Tioning	CAR-T Cell Dosing	ORR	MRD-	Survival	Other	CRS or Neuro-Toxicity	Others
Ant-BCMA CAR [63] (NIH)	1. γ-retroviral vector 2. Murine scFv 3. Co-stimulation domain: CD28 4. Culture medium: Anti-CD3 MoAb and IL-2	Phase 1 (NCT02215967)	1. 18–73 years 2. ≥3 different prior treatment. 3. BCMA expression on >50% of PC by either IHC or FCM. 4. Measurable disease 5. ECOG 0–2	1. Any anticoagulants (except aspirin) 2. Pregnant or breast-feeding 3. Active systemic infection 4. CNS involvement 5. Pregnant or lactating women	1. RRMM patients, *n* = 24. 2. Median 9.5 lines of prior therapy (range 3–19) in highest dose level group (*n* = 16). 3. High-risk cytogenetics: 40% of evaluable patients at highest dose.	Cy (300 mg/m^2^) 3 doses and Flu (30 mg/m^2^) 3 doses	Dose escalation from (0.3, 1, 3, 9) × 10^6^ CAR T cells/kg. a. 10 patients received 0.3–3 × 10^6^. b. 16 patients received 9 × 10^6^. (2 patients received 2 infusions)	(16 evaluable) ORR:81% (2 sCR, 8 VGPR, 3 PR)	100% (at 9 × 10^6^, *n* = 11), by 8-color FCM.	Median EFS: 31 weeks		(16 evaluable) 1. CRS: 15 (93.75%), including 2 grade 4, 4 grade 3, 7 grade 2, and 2 grade 1 2. 6 (38%) need vasopressor support for hypotension	(16 evaluable) 1. Grade 3–4 AEs: leikopenia (93.75%), anemia (68.75%), thrombocytopenia (62.5%)
bb2121 (Idecabtagene vicleucel) [64] (Celgene)	1. Lentivirus vector 2. Murine scFv 3. Co-stimulation domain: 4-1BB 4. Culture medium: Anti-CD3/CD28, OKT3	Phase 1 (NCT02658929)	1. ≥18 years 2. ECOG 0 or 1 3. ≥3 different prior lines of therapy 4. Measurable disease 5. ≥50% BCMA expression on PCs (IHC).	1. CNS disease 2. Inadequate organ function (heart, liver, renal) 3. Inadequate bone marrow function 4. Active systemic infection within 72 h 5. Pregnant or lactating women 6. Plasma cell leukemia	1. RRMM patients, *n* = 33. 2. Median 7 lines of prior therapy (range 3–14). All received auto-HSCT; 71% received anti-CD38 MoAb; 29% with penta-refractory 3. High-risk cytogenetics: 45%	Flu (30 mg/m^2^)/Cy (300 mg/m^2^) daily for 3 days	One infusion 3+3 design with dose levels of 5, 15, 45, 80 and 120 × 10^7^ bb2121 cells.	85% (28/33), including 3 CR and 12 sCR.	(total 18): 16/16 (100%) at 10^−4^ nucleated cell (exclude 2 no response), by NGS	1. Median DOR: 10.9 months 2. Median PFS: 11.8 months	Median time to first PR or better: 1.0 month	1. CRS: 25 (76%), grade 1 or 2 (*n* = 23, 70%), grade3 (*n* = 2, 6%) 2. Duration of CRS:5 days 3. Neurologic toxic effects: 14 (42%), including 13 grade 1–2 (39%) and 1 grade 4 (3%).	1. Grade 3–4 AEs (>10%): Neutropenia, leukopenia, anemia, thrombocytopenia, lymphopenia. 2. No DLT 3. Infection: 14 (42%), including 2 grade 3.
bb21217 [65] (bluebird bio)	1. Lentivirus vector 2. Murine scFv 3. Co-stimulation domain: 4-1BB 4. Add PI3K inhibitor bb007 in ex vivo culture	Phase 1, (CRB-402; NCT03274219)	1. ≥18 years old. 2. ECOG 0 or 1. 3. ≥3 different prior lines of therapy. 4. Measurable disease. 5. ≥50% BCMA expression on PCs (IHC).	1. CNS disease 2. Inadequate organ function(heart, liver, renal) 3. Inadequate bone marrow function 4. Active systemic infection within 72 h 5. Pregnant or lactating women	1. RRMM patients, *n* = 22 2. Median 7 lines of prior therapy (range 4–17). 3. 18 patients had prior ASCT; 7 had high-risk cytogenetics. 19 received prior daratuzumab, 13 had previously bortezomib, lenalidomide, carfilzomib, pomalidomide, and daratumumab. 4. Eleven patients had high tumor burden (≥50% bone marrow PCs) before infusion.	Flu (30 mg/m^2^)/Cy (300 mg/m^2^) daily for 3 days	One infusion with planned dose levels of 150, 450, 800, and 1200 × 10^6^ bb21217 cells.	83% (15/18), 6 of them progressed	100% (10/10), at 10^−5^ nucleated cells by NGS at month 1.			1. CRS: 13 (59.1%), including 5 Grade 1, 7 Grade 2, 1 Grade 3. All responded to supportive care. 2. 5 neurotoxicity, including 1 grade1, 2 grade 2, 1 grade 3, and 1 grade 4	1. No treatment related mortality
BCMA CAR-T [66] (HRAIN Biotechnology, Henan University)	1. γ-retrovirus vector 2. Co-stimulation domain: 4-1BB 3. Safety switch (truncated EGFR)	Phase 1 (NCT03093168)	1. 18–70 years 2. ≥3 lines of prior therapy (PI, or IMiDs, or both) 3. ≥5% BCMA expression on PCs (IHC). 4. ≥90 days after HSCT 5. ECOG 0–4	1. With CNS symptoms 2. Another malignancy 3. Active hepatitis B or C, HIV infections 4. Severe heart or respiratory diseases	1. RRMM patient, *n* = 17 (infused), 14 (evaluable for efficacy and safety)	Flu (25 mg/m^2^)/Cy (300 mg/m^2^) daily for 3 days (d-5 to -3)	One infusion of CAR-T cell: 9 × 10^6^/kg (d0)	79%, 3 sCR, 4 CR and 2 MRD- (2 VGPR)			1 sCR and 1 VGPR with the ongoing objective response ≥15 months.	1. Grade≥ 3 CRS: 1(7%) 2. Grade ≥ 3 neurotoxicity: 1(7%)	1. Grade≥ 3 non-hematologic AEs: 2 pneumonia (14%), 2 hypophosphatemia (14%), and 2 hypocalcemia (14%)
BCMA CAR T (FCARH143) [67] (Fred Hutchinson Cancer Research Center)	1. Lentivirus vector 2. Fully human scFv 3. Co-stimulation domain: 4-1BB	Phase 1 (NCT03338972)	1. ≥21 years 2. RRMM (≥10% CD138+ BM PCs, and ≥5% BCMA expression by FC). 3. ECOG 0–2 4. Measurable disease.	1. With another primary malignancy 2. Active hepatitis B or C, HIV infections 3. Uncontrolled active infection 4. CNS symptoms 5. Pregnant or lactating women	1. RRMM patient, *n* = 7 Cohort A: 10–30% MM cells in BM Cohort B: >30% MM cells in BM (Median % : 58% (20 to >80)) 2. Median 8 lines of prior therapy (range 6–11) 3. All with ≥1 high-risk cytogenetics (71% had ≥2) 4. 71% with prior ASCT, 43% with allo-SCT	Cy + Flu (d-4 to -2)	CAR T cell dosing (d0) (1:1 ratio of CD4+:CD8+ BCMA CAR T cells) Cohort A: 5 × 10^7^ Cohort B: 15 × 10^7^	100% (at 28 days)			One relapsed with BCMA- PC clone	1. No neurological toxicity 2. CRS: 6 (85.7%), all grade 2 or lower	1. No DLTs
BCMA-CAR T [68] (Huazong University)	1. Lentivirus vector 2. Murine scFv 3. Co-stimulation domain: CD28	Phase 0 (ChiCTR-OPC-16009113)	1. 18 to 70 years. 2. ECOG 0–2. 3. Adequate organ function 4. With BCMA+ PC.	1. Pregnancy and nursing females 2. Active hepatitis B or C, HIV infections 3. With mental disorders	1. 28 patients (26 RRMM, 1PCL, 1POEMS) 2. BCMA expression level Strong (≥50%): 22 patients Weak (<50%): 6 patients	Cy + Flu	5.4 to 25 × 10^6^ CAR T cells/kg	Strong: 87% (73% CR) Weak:100% (CR or VGPR)		Median DFS (strong vs weak): 296 vs 64 days3.Median OS (strong vs weak): Not defined vs 206.5 days		1.4 grade 3CRS.	1. All toxicities were fully reversible
CART-BCMA [36] (University of Pennsylvania-Norvartis)	1. Lentivirus vector 2. Fully human scFv 3. Co-stimulation domain: 4-1BB 4. Culture medium: Anti-CD3/CD28 beads and IL-2	Phase 1 (NCT02546167)	1. ≥18 years. 2.RRMM (≥3 prior treatment, or ≥2 prior regimens with double refractory to PI and IMiDs). 3. Adequate organ functions 4. ECOG 0–2 5. Measurable disease	1. Pregnant or lactating 2. Active hepatitis B or C, HIV infections 3. Active or uncontrolled infection 4. Uncontrolled medical or psychiatric diseases	1. RRMM patients, 34 consented, 29 eligible, 25 received infusion 2. Median 7 lines of prior therapy (range 3–13) 96% refractory to IMiDs and PIs 72% refractory to daratumumab 44% penta-refractory 3. 96% with at least one high-risk cytogenetics (68% del17p or *TP53* mutation) 4. Median 65% of MM cells on bone marrow biopsy 28% with extramedullary disease.	With (Cy) or without conditioning treatment	1. 3 split-dose infusions of CAR T cells (10%, 30%, 60%) 2. 3 cohorts a. 1–5 × 10^8^ CART cells b. Cy 1500m g/m^2^ + 1–5 × 10^7^ CART cells c. Cy 1500 mg/m^2^ + 1–5 × 10^8^ CART cells	(≥PR): 48%, with 55% in 5 × 10^8^ CART-BCMA cells. a. Cohort 1: 4 (44%, 1 sCR, 2 VPGR, 1 PR) b. Cohort 2: 1 (20%, 1 PR) c. Cohort 3: 7 (64%, 1 CR, 3 VGPR, 3 PR) in cohort 3.		Overall median OS: 502 days (359 days, 502 days, and not reached for cohorts 1, 2, and 3, respectively)	Detected CAR T cells: in 20 (100%) and 14 (82%) evaluable patient at 3 and 6 months post infusion.	1. CRS: 22 (88%); 8 grade 3–4 (all 1–5 × 10^8^ dose) Medium time to CRS:4 days Medium duration; 6 days Medium hospitalization: 7 days 7 (28%) received anti-IL-6 agents 2. Neurotoxicity (*n* = 8, 32%): 5 grade 1–2, 3 grade 3–4	1. All grade ≥ 3 AEs: 24 (96%) 2. Grade 3/4 SE: leukopenia (44%), neutropenia (44%), lymphopenia (36%) 3. One grade 5 AE
CART-BCMA [69] (Shenzhen Pregene Biopharma)	1. One anti-BCMA single-domain antibody derived from the alpaca, and humanized 2. Co-stimulation domain: 4-1BB	Phase 1 (NCT03661554)	1. 18–75 years 2. RRMM, BCMA+ 3. ECOG 0–2 4. Adequate organ function	1. Pregnant or lactating 2. Active hepatitis B or C, HIV infections 3. Severe infection 4. Poor organ function	1. RRMM patients, *n* = 16(infused) 2. Median 10 lines of prior therapy	Cy (300–600 mg/m^2^, d-5, -4) and Flu (30 mg/m^2^, d-5 to d-3)	One infusion of 2–10 × 10^6^ CAR cells/kg(d0)	1. 13 patients without EM lesion: 84.6% (d28), 100% (10th weeks, n = 7), including 3 sCR/CR, 1 VGPR, and 3 PR 2. Three patients with EM lesion: All PR at d28				1. 2 patients with grade3–4 CRS (0–2 in other patients)	
CART-BCMA/CART-19 [70,71] (First Affiliated Hospital of Soochow University)	1. Co-stimulation domain: OX40 and CD28 2. Lentiviral vector 3. Culture medium: Anti-CD3 beads	Phase 1/2 (NCT 03196414)	1. 18–75 years 2. CD138+ or BCMA+ RRMM 3. Adequate organ function	1. Pregnant or lactating 2. Active hepatitis B or C, HIV infections 3. Uncontrolled active infection 4. Poor organ function	1. RRMM patients, *n* = 28 2. All resistant to PIs, IMiDs, or both 3. Average of 3 (2–8) lines of prior treatment	Cy 300mg/m2 and Flu × 3 days (d-5,-4 and -3)	CART-19 (1 × 10^7^/kg on day 0) and CART-BCMA cells (40% on d1 and 60% on d2)	92.6% (88.9% PR or better), 11 CR or sCR, 8 VGPR, 5 PR and 1 MR.		Median OS: 16 months		1. CRS:100%, 19 grade 1–2, 7 grade 3, and 2 grade 4	1. Other AEs: fatigue (100%), cytopenia (100%), anemia (100%), and prolonged APTT (82.1%)
		Phase 1/2 (NCT 03455972)	1. 18–65 years old MM patients eligible for auto-HSCT. 2. High-risk MM (stage III or failed to achieve PR after prior treatment.). 3. All with BCMA >50% without CD19 expression on PCs. 4. ECOG 0–2. 5. Adequate organ function.	1. Pregnant or lactating 2. Active hepatitis B or C, HIV infections 3. Uncontrolled active infection 4. History of myocardial infarction	1. Cohort 1: 9 patients, all BCMA> 50% without CD19 expression		CART-19 (1 × 10^7^/kg on d0) and CART-BCMA cells as split-dose (40% on d1 and 60% on d2) were infused d14 to d20 after ASCT	100% (post CAR-T treatment), 3CR and 6 VGPR	37.5% after ASCT to 66.7% after CAR-T therapy			1. CRS: 100%, all grade 1–2 2. No serious CRS or neurologic complications	
CART-BCMA CTL119 [72] (Abramson Cancer Center)	1. 4-1BB co-stimulatory domain 2. Lentiviral vector		1. Phase A (PhA): MM patients responding (≥MR) to ≥3rd line therapy (or ≥2nd line if exposed to all major agents) 2. Phase B (PhB): High-risk patients.		1. 6 enrolled PhA patients were infused 2. 4 enrolled PhB patients were infused (2 CART-BCMA alone, 2 CART-BCMA + CTL119) 3. Prior lines: 1–9 4. BM PC(%): 1–91	Flu (30 mg/m^2^) + Cy (300 mg/m^2^) × 3 days	1. Phase A: CART-BCMA + CTL119 Phase B: CART-BCMA +/− CTL119 2. CAR-T infusion (5 × 10^8^ CAR+ cells in 3 divided doses, 10%, 30%, and 60%) after conditioning treatment	80%, 1 CR, 4 VGPR, 3 PR in 10 evaluable (6 PhA, 2 PhB combo, and 2 PhB mono)			1. One PhA patient died due to CNS progression before infusion. 2. All exhibited in vivo CAR-T cell expansion	(10 evaluable) CRS:80%, all grade 1–2	(10 evaluable) 8 fatigue, 8 cytopenia, 6 anemia, and 5 coagulopathy.
CT053 [73] (CARsgen Therapeutics Co.)	1. Fully human scFv2. 4-1BB co-stimulatory domain	Phase 1 (NCT03716856, NCT03302403, and NCT03380039)	1. 18–70 years old. 2. RRMM 3. BCMA+ PC (FCM or IHC) 4. Measurable disease. 5. ECOG 0–1.	1. lymphocytes transduction <10%, expansion after αCD3/CD28 costimulation <5-fold 2. Hepatitis C or HIV infections 3. Uncontrolled active infection	1. RRMM patients, *n* = 24 (All with ≥50% BCMA expression on MM cells) 2. Median 4.5 prior regimen (range 2–11) 3. 41.7% prior ASCT 4. 45.8% with EM lesions	Flu (20–25 mg/m^2^) + Cy (300–500 mg/m^2^) daily for 2–4 days	1.5 × 10^8^ CT053 cell infusion after conditioning treatment	87.5% (21/24), including 14 sCR and 5 CR	85% (17/20), ≤10^−4^ nucleated cells			1. 3 neurotoxicity (all grade 1) 2. CRS: 15 (3 grade 1, 12 grade 2), 8 received tocilizumab	1. No DLT 2. Grade ≥ 3 AEs: leukopenia, thrombocytopenia, lymphopenia.
CT103A [74] (Nanjing Iaso Biotherapeutics Co, Ltd)	1. Fully human scFv 2. With CD8a hinger and 4-1BB co-stimulatory domain 3. Lentiviral vector	Phase 0 (ChiCTR1800018137)	1. 18–70 years old. 2. BCMA+ PCs 3. Proper organ function	1. Pregnant or lactating 2. Active hepatitis B or C, HIV infections 3. Uncontrolled active infection 4. Poor organ function	1. RRMM, *n* = 16 2. Median 4 prior therapy (range 3–5), including 4 patients after murine BCMA CAR-T treatment, and 5 with EM lesions or PCL.	Cy + Flu	3 + 3 dose-escalation (3 doses at 1, 3, 6 × 10^6^/kg)	1. 100% (6 CR/sCR) within first 2 weeks. 2. 3 sCR and 1 VGPR in 4 prior BCMA CAR-T cell treated patients	100% in all 15 patients, ≤10^−4^ nucleated cells by FCM.		CT103A cells detectable in 12/16 patients, at the last evaluation	1. All developed CRS (10 grade 1–2, 5 grade 3, 1 grade 4)	
JCARH125 [75] (Juno Therapeutics, Inc.)	1 Fully human scFv 2. Co-stimulation domain: 4-1BB 3. Lenti-viral vector	Phase 1/2 (EVOLVE; NCT 03430011)	1. ≥18 years old 2. RRMM (≥3 prior regimens, including PI, IMiD, anti-CD38 MoAb, and auto-HSCT). 3. ECOG 0–1 4. Adequate renal, BM, liver, lung, and heart function 5. Measurable disease	1. EM lesion, PCL, WM, or POEMS syndrome. 2. CNS involvement by malignancy 3. Untreated or active infection 4. Poor heart function	1. RRMM patients (19 enrolled, 13 treated) Initial 8 patients 1. Median 10 lines of prior therapy (range 4–15), including 50% refractory to bortezomib, carfilzomib, lenalidomide, pomalidomide and an anti-CD38 mAb. 2. 88% had prior ASCT	Flu (30 mg/m^2^)/Cy (300 mg/m^2^) daily for 3 days	One infusion of JCARH125 (2 dose levels: 50 and 150 × 10^6^ CAR+ T cells)	Evaluable patients (*n* = 3), 1PR, 2 sCRs Unconfirmed patients (*n* = 5): 1 CR, 2 VGPR, 1 PR, 1 MR				(8 evaluable) 1. CRS: 6 (75%), all grade 1or 2 Median onset of CRS: 9 days (range 4 – 10) Median duration of CRS: 4.5 days (range 2 – 19 days) 2. Neurotoxicity: 3 (2 grade1, 1 grade 3)	
LCAR-B38M [76,77] and [78,79] (Nanjing Legend Biotech Co)	1. Lentivirus vector 2. Bispecific anti-BCMA variable fragments of llama heavy-chain antibodies 3. Co-stimulation domain: 4-1BB 4. Culture medium: IL-2	Phase 1/2 (LEGEND-2; NCT 03090659)	1. 18–80 years 2. RRMM (≥3 prior regimens) 3. BCMA+ PC (FCM or IHC)	1. Pregnant or lactating 2. Active hepatitis B or C, HIV infections 3. Uncontrolled medical illness	(One of four centers) 1. RRMM patients, *n* = 57 2. Median 3 prior regimens (range1–9), including prior PIs (68%), IMiDs (86%), and both (60%)	3 doses of Cy 300 mg/m^2^	Five days after Cy, LCAR-B38M CAR T cells (median cell dose = 0.5 × 10^6^ cells/kg, [range, 0.07–2.1 × 10^6^]), split into in 3 infusions (20, 30, and 50% of total dose) given over 7 days.	≥PR: 88% (50/57), including 42 CR (39 MRD-), 2 VGPR, 6 PR.	92.8% (39/42) in CR patients, by 8-color FCM	1. Median DOR: 22 months 2 Median PFS: 20 months (all patients); 28 months (MRD- patients). 3. The median OS: NR	Medium time to response: 1.2 month	1. CRS: 51 (90%), grade 1 (47%), grade 2 (35%); grade 3 (7%, *n* = 4). Medium time to CRS; 9 days 2. Neurotoxicity: 1 (grade 1), dosed at 1.0 × 10^6^ CAR+ T cells/kg	1. AE reported in all patients. Pyrexia (91%), thrombocytopenia (49%), and leukopenia (47%) 2. Grade ≥3 AE:37 (65%), leukopenia (30%), thrombocytopenia (23%), and increased aspartate aminotransferase (21%). 3. One grade 5 AE: pulmonary embolism
		Phase 1/2 (LEGEND-2; NCT 03090659)	1. 18–80 years 2. RRMM (≥3 prior regimens) 3. BCMA+ PC (FCM or IHC)	1. Pregnant or lactating 2. Active hepatitis B or C, HIV infections 3. Uncontrolled medical illness	(The other three centers) 1. RRMM patients, *n* = 17 2. ≥3 prior regimens (range3-11), including prior PIs (88%), IMiDs (82%), and both (71%), ASCT (47%) 3.Five patients with EM	Cy 250 mg/m^2^ + Flu 25 mg/m^2^ for 3 days (*n* = 8) or Cy 300 mg/m^2^ for 3 days (*n* = 9).	LCAR-B38M cell infusion 5d after the start of the conditioning regimen. (3 infusions in Cy + Flu vs 1 infusion in Cy group) Mean dose: 0.7 × 10^6^ (range, 0.2–1.5 × 10^6^ cell/kg)	1. 88% (15/17), including 14 CR and 1 VGPR 2. With EM lesion: 100%, including 3 sCR, 1VGPR, and 1 MR	100% in all CR patients (8-color FCM)	1. Median PFS: 12 (all) and 18 (MRD-) months 2. Median OS: NR (all and MRD-)	Cy + Flu group had better PFS and lower relapse rate	1. CRS:100%, including 10 grade 1/2, 6 grade 3, and 1 grade 5. 9 patients received IL-6R inhibitor treatment. 2. No neurotoxicity	1. AE reported in all patients. Pyrexia (100%), cytopenia (82%), impaired liver function (100%) 2. Tumor lysis syndrome: 3(18%)
MCARH171 [80] (Memorial Sloan Kettering Cancer Center)	1. Human derived anti-BCMA scFv 2. Co-stimulation domain: 4-1BB 3. Retroviral vector 4. Safety switch (truncated EGFR) 5. Culture medium: Phytohemagglutinin or anti-CD3/CD28 beads and IL-2	Phase 1	1.≥18 years. 2. RRMM (≥2 prior regimens including an IMiD and a PI) 3. Adequate organ function	1. Poor performance 2. Poor organ function 3. HIV or active hepatitis B or hepatitis C infection	1. RRMM patients, *n* = 11 2. Median 6 lines of prior therapy (range 4–14), all received IMiD, anti-CD38 MoAb, and ASCT 3. 82% with high-risk cytogenetics	(1) Cy 3000 mg/m^2^ single dose or (2) Flu 30 mg/m^2^ daily and Cy 300 mg/m^2^ daily for 3 days	1-2 divided doses of MCARH171 with 4 dose levels (1) 72 × 10^6^, (2) 137 × 10^6^, (3) 475 × 10^6^, (4) 818 × 10^6^ viable CAR T cells	1. ORR:64% 2. 100% ORR observed in 5 patients received higher doses (≥ 450 X10^6^)		Median DOR:106 days		(10 evaluable) 1. CRS: 6 (60%), 4 grade 1–2, and 2 grade 3. 2. No grade ≥3 neurotoxicity	(10 evaluable) 1. No DLTs
JNJ-68284528 [81] (Janssen)	1. Lentivirus vector 2. Bispecific anti-BCMA variable fragments of llama heavy-chain antibodies 3. Co-stimulation domain: 4-1BB 4. Culture medium: IL-2 (Identical to LCAR-B38M)	Phase 1b/2 (CARTITUDE-1/MMY2001; NCT03548207)	1. ≥18 years old 2. RRMM (≥3 prior regimens or double refractory to a PI and IMiD, and received an anti-CD38 MoAb) 3. Measurable disease 4. ECOG 0–1	1. Previous CAR-T treatment (+) 2. Previous anti-BCMA treatment (+) 3. Poor heart function 4. CNS MM involvement	1. RRMM patient, *n* = 25 (infused) 2. Median prior lines of treatment : 5 (range 3–16) 3. 88% triple-refractory to a PI, IMiD, and anti-CD38 antibody, 72% penta-exposed, and 36% penta-refractory	Flu (30 mg/m^2^)/Cy (300 mg/m^2^) daily for 3 days	One infusion of JNJ-4528 (target 0.5–1 × 10^6^ /kg) 5–7 days after conditioning treatment	(21 evaluable) 1. ORR:91%, 4 sCR, 2CR, 7 VGPR, and 6PRs	(15 evaluable) 1. 10 MRD- at the 10^−5^ level, 2 at the 10^−4^ level, and 3 had unidentified clones.			1. 80% of patients had grade 1–2 CRS, with 1 grade 3 and 1 grade 5. 2. CRS events occurring at a median of 7 days (range 2–12) post-infusion with a median duration of 3 days (range 1–60). 4. Tocilizumab and steroid used in 91% and 27% of patients (*n* = 22).	1. Treatment related AE: CRS (88%), neutropenia (80%), anemia (76%), and thrombocytopenia (72%) 2. Grade ≥3 AEs: neutropenia (76%), thrombocytopenia (60%), and anemia (48%)
P-BCMA-101 [82] (Poseida Therapeutics, Inc.)	1. Centyrin-based binding domain (small, fully human) 2. CD3ζ/4-1BB signaling domain 3. *In vitro* transcribed mRNA and plasmid DNA 4. Safety switch (truncated EGFR) 5. Tscm phenotype	Phase 1 (NCT 03288493)	1. ≥18 years old. 2. RRMM (received PI and IMiDs) 3. Measurable disease 4. Adequate organ function	1. Pregnant or lactating 2. Active hepatitis B or C, HIV infections 3. Uncontrolled medical illness 4. With PCL, WM, or POEMS syndrome 5. Active second malignancy	1. RRMM patients, *n* = 12 2. Rang of prior lines: 3–9 3. 100% refractory to PI, IMiD, and daratumumab 4. High-risk cytogenetics: 64%	Flu (30 mg/m^2^)/Cy (300 mg/m^2^) daily for 3 days	1. 1 infusion of P-BCMA-101. 2. 3 + 3 design with planned dose levels of 48, 50, 55, 118, 122, 124, 143, 155, 164, 238, 324 and 430 × 10^6^ CAR T cells.	1. 66.7%(*n* = 3), 1 PR and 1 near CR 2. Yet evaluable patients (*n* = 6): 1 sCR, 1VGPR, and 3PRs				1. CRS:1 (grade 2) 2. No neurotoxicity	1. No unexpected/off-target toxicities related to treatment.

The inclusion or exclusion criteria were summarized from published articles or ClinicalTrials.gov website.

**Table 2:** Summary clinical study results of anti-BCMA ADC, BiTE, and bispecific antibodies.

Name (Sponsor)	Basic Data of Participant	Protocol	Results and Efficacy	Adverse Event (AE)
GSK2857916 (Belantamab mafodotin) [85,86,87] (GlaxoSmithKline)	1. Phase 1 trial (BMA117159 /DREAMM-1, NCT 02064387) 2. RRMM patients Total: 73 (Part 1: 38; Part 2: 35) 3. Prior treatment Part 1: 29/38 (76%) ≥ 5 prior treatment lines Part 2: 20/35 (57%) ≥ 5 prior treatment lines, 97% refractory to PIs, 91% to IMiDs, 37% to daratumumab	(Part 1, dose escalation) IV 1 h ever 3 weeks Dose: 0.03, 0.06, 0.12, 0.24, 0.48, 0.96, 1.92, 2.5, 3.4, and 4.6 mg/kg (Part 2, dose expansion) IV 1 h ever 3 weeks Dose; 3.4 mg/kg	(Part 2) 1. ORR: 60% (21/35), including 1 sCRs, 2 CRs, 15 VGPRs, and 3 PRs. 2. Median time to response: 1.4 months 3. Median PFS: 7.9 months	(Part 1) No DLT was identified (Part 2) 1. IRR: 8 (23%), mainly grade 1 or 2 (*n* = 5) 2. Corneal event: 22 (63%), mainly grade 1 or 2 (*n* = 19), 3 grade 3. 3. Thrombocytopenia: 20 (57%), 12 grade 3–4
	1. Phase 2, two-armed, randomized trial (DREAMM-2, NCT 03525678) 2. 293 RRMM (screened), 196 receive treatment 3. All refractory to IMiDs, PIs, and previously received an anti-CD38 MoAb	Two cohorts (IV 30 mins or longer) 1. 2.5 mg/kg *(n* = 97) 2. 3.4 mg/kg (*n* = 99) Treatment every 3 weeks until PD or unacceptable toxicity	1. ORR: 32.7% (64/196), 30 in the 2.5 mg/kg cohort and 34 in the 3.4 mg/kg cohort, with 18 and 29 achieving VGPR or better (CR or sCR) 2. DOR: NR (median follow-up of 6.3 and 6.9 months) 3. Probability of having a DOR≥ 4 months: 78% and 87% (2.5 and 3.4 mg/kg cohort) 4. Median PFS: 2.9 and 4.9 months	Safety population, *n* = 194 1. 93 (98%) of 95 in the 2.5 mg/kg cohort and 99 (100%) of 99 in the 3.4 mg/kg cohort had at least one AE. 2. Keratopathy is the most common Grade 1–2 and 3–4 AE. a. Dose delays for keratopathy started at week 4 in both cohorts b. Dose reductions started at 13 and 4 weeks (2.5 and 3.4 mg/kg cohort). c. Median time to treatment re-initiation: 83 and 63 days (2.5 and 3.4 mg/kg cohort). 3. Other common Grade 3-4 AE: thrombocytopenia (19/33) and anemia (19/25) 4. Serious AE (38/47). 5. Two treatment related death
BI 836909/AMG 420 [88] (Amgen)	1. Phase 1 trial (NCT02514239) 2. RRMM patients (total: 35) 3. ≥2 prior treatment lines, including PI and IMiDs	6-week cycles of (1 cycle = 4 weeks continuous IV infusion, 2 weeks off). Single-patient cohorts [0.2–1.6 µg/day (d)] followed by cohorts of 3–6 patients (3.2–800 µg/d)	1. 6 CRs (1 each at 6.5, 100, and 200 µg/d, and 3 at 400 µg/d) 2. All patients at 400 µg/d (3/3) had MRD negative CRs 3. Dose confirmation cohort (400 µg/d): 2 PR (2/3) 4. Objective response rate at 400 µg/d: 83% (5/6)	1. Serious AEs (*n* = 17, 49%), including 10 infection, 3 CRS, and 1 each of peripheral polyneuropathy, cardiac failure, edema, pyrexia, biliary obstruction, and renal failure. 2. CRS (*n* = 3, 2 grade 1 and 1 grade 3)
CC-93269 [89] (Celgene)	1. Phase 1, dose-finding study (CC-93269-MM-001; NCT03486067) 2. RRMM patients (total:19), with ≥3 prior treatment lines 3. Medium 6 lines of prior therapy (range 3–12 lines) 73.7% ASCT 10.5% allogenic stem cell transplantation 100% lenalidomide and 84.2% pomalidomide 100% bortezomib and 84.2% carfilzomib 94.7% daratumumab	Cycles 1–3: IV over 2 h on Days 1, 8, 15, and 22 Cycles 4–6 Days 1 and 15 Cycle 7 and beyond Day 1 (all in 28-day cycles) Doses ranged from 0.15 to 10 mg	1. <6 mg (*n* = 7), response:0 2. ≥6 mg(*n* = 12), response: 10, (4 sCR or CR, 3 VGPR, 3 PR), 9 MRD-	1. Grade 3–4 treatment AE: 15 (78.9%), including 10 neutropenia, 8 anemia, 5 infections, and 4 thrombocytopenia 2. CRS: 17 (89.5%), grade 1 (*n* = 11 (57.9%) or grade 2 (*n* = 5, 26.3%)
PF-06863135 [90] (Pfizer)	1. Phase 1 trial (NCT03269136) 2. Relapsed (*n* = 8) and refractory MM patients (*n* = 9). 3. Median prior lines of treatment: 11.5 (All previously treated with a PI, an IMiD, and an anti-CD38 MoAb) 4. 5 (29%) patients had received prior BCMA-targeted therapy (CAR-T or BiTE)	Once weekly, non-continuous, IV infusion in 6 dose-escalation groups	16 evaluable 1. 1 MR and 6 SD 2. Clinical benefit: 41%	1. 10 patients experienced treatment AE, mostly grade 1–2, including CRS (24%), thrombocytopenia (24%), anemia (18%), and pyrexia (18%) 2. Three grade 3 3. No grade 4–5 AE 4. One DLT in a patient previously treated with BCMA CAR-T.

ASCT, autologous stem cell transplant; Cy, cyclophosphamide; CR, compete response; CRS, cytokine releasing syndrome; DLT, dose-limiting toxicity; DOR, duration of response; EGFR, epidermal growth factor receptor; EM, extramedullary; Flu, fludarabine; IRR, infusion related reaction; MoAb, monoclonal antibody; MTD, maximum tolerated dose; MR, minimal response; MRD, minimal residual disease; MRD-, MRD-negative; NR, not reached; ORR, overall response rate; OS, overall survival; PFS, progression-free survival; PR, partial response; PRES, posterior reversible encephalopathy syndrome; RRMM, relapsed and refractory multiple myeloma; SD, stable disease; URI, upper airway infection; UTI, urinary tract infection; VGPR, very good partial response.

**Table 3:** Comparison of BCMA-based immunotherapeutic agents in an RRMM setting.

Perspective	CAR-T cell therapy	BiTE/Bispecific Antibodies	Antibody-Drug Conjugate
**Strength**	1. Most clinical data support clinical efficacy 2. High response/MTD- rate in RRMM patients received multiple lines of prior treatment 3. May be effective for extramedullary disease 4. Development of long-term anti-tumor immunity	1. “Off-the shell” products, no delay in treatment 2. Clinical benefit observed in RRMM patients 3. No lymphodepletion treatment required	1. “Off-the shell” products, no delay in treatment 2. Clinical benefit observed in RRMM patients 3. Potent anti-tumor activity of payload 4. Clinical efficacy doesn’t rely on host immune function status
**Challenges**	1. Long and labor-intensive manufacturing process 2. Require treatment at a specialized center 3. High CRS and neurotoxicity rate 4. Toxicity of lymphodepletion therapy 5. Clinical activity relies on adequate number and function of collected patients T cells 6. High treatment cost	1. Less data in heavily pretreated patients, may be less effective in this subgroup 2. CRS and neurotoxicity 3. Short half-life, need continuous infusion 4. Clinical activity relies on adequate number and function of collected patient’s effector cells 5. Hight treatment cost	1. High serum level of sBCMA may affect clinical efficacy 2. Payload related toxicity 3. Multiple treatment leads to increased cost.
**Prospects**	1. Allogeneic CAR-T cells 2. Faster manufacturing protocol 3. Structure modification to reduce toxicity, i.e., safety switch	1. Half-life extended products 2. Structure (variable) modification to increase binding affinity for tumor associated antigen	1. New design of antibody to increased binding affinity for tumor-associated antigen 2. New payload with novel anti-tumor mechanism and better safety profile 3. Linker optimization to reduce off-target effect (slow deconjugation)
